# Human Puumala hantavirus infection in northern Sweden; increased seroprevalence and association to risk and health factors

**DOI:** 10.1186/s12879-016-1879-2

**Published:** 2016-10-13

**Authors:** Kristina Bergstedt Oscarsson, Alette Brorstad, Maria Baudin, Anne Lindberg, Annika Forssén, Magnus Evander, Marie Eriksson, Clas Ahlm

**Affiliations:** 1 Department of Clinical Microbiology, Infectious Diseases, Umeå University, SE-901 85 Umeå, Sweden; 2 Department of Clinical Microbiology, Virology, Umeå University, Umeå, Sweden; 3 Department of Public Health and Clinical Medicine, Medicine, Umeå University, Umeå, Sweden; 4 Department of Public Health and Clinical Medicine, Family Medicine, Umeå University, Umeå, Sweden; 5 Department of Statistics, Umeå School of Business and Economics, Umeå University, Umeå, Sweden

**Keywords:** Emerging infection, Hantavirus, Haemhorrhagic fever with renal syndrome, Puumala, seroepidemiology, Risk factors

## Abstract

**Background:**

The rodent borne Puumala hantavirus (PUUV) causes haemorrhagic fever with renal syndrome in central and northern Europe. The number of cases has increased and northern Sweden has experienced large outbreaks in 1998 and 2006–2007 which raised questions regarding the level of immunity in the human population.

**Methods:**

A randomly selected population aged between 25 and 74 years from northern Sweden were invited during 2009 to participate in a WHO project for monitoring of trends and determinants in cardiovascular disease. Health and risk factors were evaluated and sera from 1,600 participants were available for analysis for specific PUUV IgG antibodies using a recombinant PUUV nucleocapsid protein ELISA.

**Results:**

The overall seroprevalence in the investigated population was 13.4 %, which is a 50 % increase compared to a similar study only two decades previously. The prevalence of PUUV IgG increased with age, and among 65–75 years it was 22 %. More men (15.3 %) than women (11.4 %) were seropositive (*p* < 0.05). The identified risk factors were smoking (OR = 1.67), living in rural areas (OR = 1.92), and owning farmland or forest (OR = 2.44). No associations were found between previous PUUV exposure and chronic lung disease, diabetes, hypertension, renal dysfunction, stroke or myocardial infarction.

**Conclusions:**

PUUV is a common infection in northern Sweden and there is a high life time risk to acquire PUUV infection in endemic areas. Certain risk factors as living in rural areas and smoking were identified. Groups with increased risk should be targeted for future vaccination when available, and should also be informed about appropriate protection from rodent secreta.

**Electronic supplementary material:**

The online version of this article (doi:10.1186/s12879-016-1879-2) contains supplementary material, which is available to authorized users.

## Background

Hantaviruses of the *Bunyaviridae* family are globally spread and each human pathogenic hantavirus is carried by different rodents [1]. The virus is mostly transmitted to humans by inhalation of virus particles present in rodent secreta and cause haemorrhagic fever with renal syndrome (HFRS) or hantavirus pulmonary syndrome (HPS) depending on the hantavirus species. HFRS in Asia is caused by Hantaan and Seoul viruses, and in central and northern Europe Puumala virus (PUUV) is the endemic hantavirus whereas Dobrava virus is found in the Balkans [1]. In the Americas, Andes, Sin Nombre and related viruses cause HPS which is a more severe infection. The prodrome in HPS is similar to HFRS, however the disease often proceeds into cardiopulmonary failure, with a case-fatality rate up to 40 % [1]. In Scandinavia, the only human pathogenic hantavirus recognized so far is PUUV which causes a less severe HFRS, also denoted *nephropathia epidemica* characterized by abrupt onset of fever, resembling influenza with myalgia, headache, fatigue and varying degrees of renal impairment [2]. In addition, abdominal pain, nausea, vomiting, and backache may be present [3]. The seroprevalence has previously been estimated at 9 % in northern Sweden [4]. In Sweden, PUUV infection is a notifiable disease and the reported incidence rate varies from 20 to 313 per 100,000 persons each year, but the real figure is considered to be seven to eight times higher [5, 6]. Hantavirus antibodies have been found more than 50 years after a diagnosed PUUV infection, and life-long immunity is suggested [7]. The primary reservoir is bank voles, *Myodes glareolus*, and the incidence of PUUV infection is correlated to the size of the bank vole populations [1]. Person-to-person transmission has been shown for the South American Andes hantavirus [8]. In Europe, transmission between humans has not been reported, although PUUV has been detected in human saliva [9]. Known risk factors for contracting the infection are agricultural work and activities in peridomestic areas such as cleaning of sheds or summer cottages, wood handling and smoking [2, 10–13]. The case-fatality rate is less than 1 % [7, 11] and PUUV infection has generally a favourable prognosis. However, cardiopulmonary involvement, increased risk for myocardial infarction, stroke, and hormonal deficiencies may occur during and after the infection [14–16].

Furthermore, several studies indicate possible association to hypertension and renal impairment [17, 18] although the findings have been contradictory [19].

During the winter 2006–2007 a very large outbreak of PUUV infection occurred in northern Sweden [6]. This sudden outbreak raised questions regarding the level of immunity against PUUV among the population, risk factors for disease and health consequences.

The aims of the present study were; to define the current seroprevalence of PUUV infection in northern Sweden; to define risk factors for infection, and link these findings to demographic and health data in a large, randomly selected and stratified population sample. In addition, we investigated potential associations between previous PUUV infection and cardiovascular risk factors and/or chronic diseases.

## Methods

### Study site and population

The MONICA project, “Multinational Monitoring of Trends and Determinants in Cardiovascular Disease” was initiated by the World Health Organisation (WHO) in 1982. The purpose was to study levels, trends and risk factors of stroke and coronary disease globally [20]. In northern Sweden, the counties of Norrbotten and Västerbotten, with a combined population of approximately 500,000 inhabitants, joined the MONICA project in 1985 [21]. When the official WHO assignment was concluded in 1994, the project continued as the Northern Sweden MONICA study.

During spring 2009, 2,500 individuals from the counties of Norrbotten and Västerbotten were randomly selected. 250 males and 250 females from each age group 25–34, 25–44, 45–54, 55–64 and 65–74 were invited to participate. After signing a written consent, they received a free health check including measuring of arterial blood pressure, waist − hip ratio and Body Mass Index (BMI). Levels of S − Creatinine were measured.

A questionnaire regarding quality of life, previous and present illness, social support, health, work, physical activity, diet and use of tobacco was answered.

Subjects not responding to the study invitation (*n* = 771), were contacted and asked to complete a basic questionnaire of which 485 answered. They were on average younger (46.7 vs. 50.8 years, *p* < 0.001), less likely to being married/cohabitant (63.6 % vs. 73.9 %, *p* < 0.001), and less likely to have a university education (23.9 % vs. 29.9 %, *p* = 0.010) than the participants. They more often reported themselves as diabetics (7.7 % vs. 4.8 %, *p* = 0.013), and as smokers (17.5 % vs. 11.0 %, *p* < 0.001). BMI, based on self − reported height and weight, was equivalent to the BMI measured in participants (data not shown). Treatment with hypertensive medication (22.7 % vs. 19.3 %, *p* = 0.106) and lipid − lowering drugs (9.1 % vs. 12.4 %, *p* = 0.052) did not differ significantly between participants and non − participants.

### Definitions

Rural areas were defined as communities having less than 1,000 inhabitants. The municipalities of the counties of Västerbotten and Norrbotten were divided into inland or coastal. The participants stated their highest educational level as primary school (up to nine years of school), secondary school (ten to twelve years of school) or university studies (higher education). The participants were asked to describe their main occupation, descriptions later classified according to the “Nordic standard of occupational classification” of 1985 [22]. In order to identify possible occupational risks for hantavirus infection, occupations with similar assumed exposure were grouped together as; 1) office personnel; 2) teaching, clergy, police and military; 3) healthcare workers; 4) construction, wood industry and plumbers; 5) engineers; 6) transportation, trade and service; 7) industry; 8) agricultural work. In addition, it was asked whether the participants were owners of farmland or forests.

Smoking habits were classified as regular smoker, smoking a least one cigarette a day, or non-smokers [23]. Individuals were asked to rank their own present health in one of five categories; 1) Very well, 2) Quite well, 3) In between, 4) Quite bad, 5) Bad. In the analysis we merged categories one and two as “satisfying health” and categories three until five as “unsatisfying health”.

Blood pressure was measured in a standardized manner, mean value of two measurements, taken with Hawksley’s random − zero sphygmomanometer. Hypertension was defined as a mean systolic blood pressure ≥ 140 mmHg or a mean diastolic blood pressure ≥ 90 mmHg or use of hypertensive medication. Questions about asthma, chronic obstructive pulmonary disease, diabetes, stroke and hospitalisation for acute myocardial infarction were answered with yes or no. Renal affection was measured according to the CKD − EPI (Chronic Kidney Disease Epidemiology Collaboration) equation, glomerular filtration rate (GFR) = 141 × min(Scr/κ,1)^α^ × max(Scr/κ, 1)^–1.209^ × 0.993^Age^ × 1.018 (if female) × 1.159 (if black) (Scr is serum creatinine (mg/dL), κ is 0.7 for females and 0.9 for males, α is −0.329 for females and −0.411 for males, min indicates the minimum of Scr/κ or 1, and max indicates the maximum of Scr/κ or 1.

### Laboratory methods/analysis

To perform the indirect enzyme − linked immunosorbent assay (ELISA), 96 − well microtiter plates (NUNC Immuno plates) were coated with PUUV nucleocapsid protein (diluted 1/600 in PBS) produced in *Escherichia coli* as previously described [24]. The plates were stored at −80 °C until usage when they were thawed, washed once with PBS − 0.05 % Tween (PBS − T), blocked with blocking/dilution buffer (1 % non − fat dry milk in PBS − 0.05 % Tween 20) and then washed again with PBS − T. Samples and controls were diluted to 1/420 in dilution buffer and 190 μl was added to the blocked plate. The plates were then either incubated at 4 °C overnight or at 37 °C for 2 h. After incubation, the plates were washed four times with PBS − T and the secondary antibody (goat α − human IgG conjugated with alkaline phosphatase (Invitrogen AHI0305) diluted to 1/6,000 in dilution buffer was added and incubated for 1 h at 37 °C. One tablet of 4 − Nitrophenyl phosphate disodium salt hexahydrate, pNPP (Sigma S0942 − 200TAB) was dissolved in 5 ml diethanol amine buffer and 100 μl was added to the plates after 4 washes with PBS − T. The plates were incubated at 37 °C for 30 min and the reaction was stopped with 50 μl 3 M NaOH. The absorbance at 405 nm (OD) was measured.

For cut − off calculations, 106 sera from children under the age of 5 years old were analysed as above. From these results the cut off was calculated as mean + 3 standard deviations (SD), corresponding to an OD value of 0.240.

### Statistical analysis

Binary logistic regression was used for simple group comparisons. To simultaneously analyse several possible risk factors associated with hantavirus infection (including sex, age, education, smoking, occupation, urban or rural environment, and coastal or inland living area), multiple logistic regression was used. Outcome was presented by odds ratios (OR) with corresponding 95 % confidence intervals (CI). The level of statistical significance was set to 0.05. Statistical analyses were carried out using SPSS 18.1.

Non-responders and responders were compared using t-test for continuous variables and *X* ^2^-test for categorical variables.

## Results

Of the 2,500 men and women who were invited to the survey, 1,729 (69.2 %) participated. Of these, blood samples were available for testing in 1600 individuals. The overall prevalence of PUUV IgG antibodies in serum was 13.4 % (*n* = 214), and significantly higher in men than in women (15.3 % vs 11.4 %, *p* < 0.05). There was an increasing seropositivity associated to older age for both sexes. In the age group 65–75 years, the prevalence was 22 % (Table 1). Significantly higher seroprevalence was found in rural compared to urban areas (22.8 % vs. 10.9 %), and a living in inland compared to coastal municipalities was associated with higher prevalence (16.1 % vs. 12.2 %) (Table 1).

**Table 1:** Previous PUUV infection in relation to sex, age group, environment, and region

Variable (Valid observations)		Subjects within each group	Seropositity for PUUV	Multiple logistic regression	
		n (% of study population)	n (%)	Odds ratio (OR)	95 % CI^a^ of OR
Total study population		1600 (100 %)	214 (13.4 %)	-	-
Sex	Women	789 (49.3 %)	90 (11.4 %)	1	-
	**Men**	**811 (50.7 %)**	**124 (15.3 %)**	**1.40**	**1.05 − 1.88**
Age	25 − 34 years	246 (15.4 %)	12 (4.9 %)	1	-
	35 − 44 years	326 (20.4 %)	22 (6.7 %)	1.41	0.68 − 2.91
	**45 − 54 years**	**313 (19.6 %)**	**43 (13.7 %)**	**3.11**	**1.60 − 6.03**
	**55 − 64 years**	**354 (22.1 %)**	**57 (16.1 %)**	**3.74**	**1.96 − 7.14**
	**65 − 74 years**	**361 (22.6 %)**	**80 (22.2 %)**	**5.55**	**2.95 − 10.44**
Environment^b^ (*n* = 1560)	Urban	1253 (80.3 %)	137 (10.9 %)	1	-
	**Rural**	**307 (19.7 %)**	**70 (22.8 %)**	**1.92**	**1.38 − 2.67**
Region (*n* = 1600)	Coast	1115 (69.7 %)	136 (12.2 %)	1	-
	Inland	485 (30.3 %)	78 (16.1 %)	1.29	0.95 − 1.75

^a^Confidence interval. ^b^40 subjects could not be classified according to this variable. Significant results are shown in bold formatting

There was a significantly higher seroprevalence in smokers than in non − smokers (20.6 % vs. 12.3 %) (Table 2). There were a small but not significant difference in seroprevalence in participants with higher compared to lower education level (9.5 % vs. 15 %) (Table 2). Regarding occupational risks, agricultural workers showed a prevalence of 31 %, compared to office personnel 9 % (*p* < 0.001). However, this difference was not significant in the multiple regression analyses when adjusted for sex, age, education, smoking, and living area (Table 2).

**Table 2:** Seroprevalence towards PUUV in relation to smoking, education, farming and other occupations

Variable (Valid observations)		Subjects within each group	Seropositity for PUUV	Multiple logistic regression	
		n (% of study population)	n (%)	Odds ratio (OR)	95 % CI^a^ of OR
Smoking (*n* = 1586)	No	1411 (89.0 %)	174 (12.3 %)	1	-
	**Yes**	**175 (11.0 %)**	**36 (20.6 %)**	**1.67**	**1.11 − 2.52**
Level of education (*n* = 1555)	Primary and secondary school	1128 (70.5 %)	169 (15 %)	1	-
	University education	427 (39.5 %)	45 (9.5 %)	0.80	0.58 − 1.09
Owner of farm/forest (*n* = 1600)	No	1545 (96.6 %)	194 (12.6 %)	1	-
	**Yes**	**55 (3.4 %)**	**20 (36.4 %)**	**2.44**	**1.30–4.59**
Occupation (*n* = 1410)	Office personnel	268 (19.0 %)	26 (9.7 %)	1	-
	Teaching, clergy, police, military	207 (14.7 %)	23 (11.1 %)	1.25	0.66–2.37
	Healthcare workers	238 (16.9 %)	30 (13.7 %)	1.53	0.86–2.72
	Construction, wood industry, plumbery	73 (5.2 %)	10 (13.7 %)	1.42	0.61–3.30
	Engineers	166 (11.8 %)	25 (15.1 %)	1.68	0.89–3.18
	Transportation, trade and service	279 (19.8 %)	36 (12.9 %)	1.23	0.70–2.16
	Industry	143 (10.1 %)	26 (18.2 %)	1.79	0.94–3.39
	Agricultural work	36 (2.6 %)	11 (30.6 %)	2.47	0.99–6.15

^a^Confidence interval. Significant results are shown in bold formatting

The highest prevalence of PUUV antibodies was found in farm- or forest owners; 36 %, and the risk was significantly increased; OR 2.44 (95 % 1.30–4.59) when compared with non-farm- or forest owners.

No relation was found between the presence of PUUV antibodies and hypertension, renal impairment, or self-reported asthma, chronic obstructive pulmonary disease, diabetes, previous acute myocardial infarction or cerebral insult (Additional file 1: Table S1). Also, there was no correlation between positive hantavirus serology and present unsatisfying self − assessed global health (Additional file 1: Table S1).

## Discussion

The prevalence of PUUV antibodies found in this population based cohort was 13.4 % which is higher than previously reported from the same area. A similar study in Northern Sweden in 1990, established a seroprevalence of 8.9 % [4]. The increased seroprevalence is consistent with a reported increased incidence of PUUV infection both in Sweden and Finland and several outbreaks since 1990 as well as the large outbreak 2007 in Sweden [6, 7]. This indicates that PUUV infection is increasing in Fennoscandia and should be recognized as an important viral infection in endemic areas. Our data revealed that every fourth man and every fifth woman in Northern Sweden had experienced infection with PUUV at age 65–74 years. This finding indeed confirmed a high life time risk for both men and women to acquire PUUV infection in endemic areas.

The high seroprevalence confirmed the notion that the infection is under-diagnosed and most cases are not reported. This could partly be explained by the various different, unspecific symptoms of PUUV infection that could make diagnosis difficult [3]. Also, many participants may not have attended health care facilities since symptoms could be mild and be similar to influenza.

Notably, smokers in our study had an increased risk of previous PUUV infection with an odds ratio of 1.7 (CI 1.1 − 2.5), even after adjusting for age, sex, level of education, and living area. This finding is in accordance with previous case-control studies from Finland and Sweden [12, 13]. It is believed that smokers have an increased risk to catch and to become more severely ill from respiratory, viral, and bacterial infections [25]. If the increased risk for smokers to get PUUV infection is connected to immunological mechanisms, the state of the respiratory tract, particles in cigarette smoke, or is behaviourally related remains unclear.

Furthermore, in accordance with previous findings, there was a strong relation between living in rural, forested areas or close to farms and being at risk of hantavirus infection [12, 13]. Risk factors like living in near contact with rodents, wood handling, farming, forestry work and cleaning sheds and shelters are more likely in rural areas [13]. Our results support previous studies where an increased risk of hantavirus infection to those engaged in farming and forestry [5, 10].

There was a non-significant higher seroprevalence of those in agricultural work and industrial occupations potentially due to more rodent contact. PUUV infection is more often diagnosed in males and is suggested to reflect increased exposure in male’s recreational and occupational activities [7]. Men are more often farm- and forest-owners, among which recognition of symptoms as well as notification is well established [7, 3]. In the present study there was a significantly higher seroprevalence in men compared to women. A previous study showed similar trend but no statistical significant difference in prevalence of specific IgG antibodies between males and females [5]. The high seroprevalence in women can be attributed to women’s engagement in non-occupational “peridomestic” work, such as cleaning of sheds and summer cottages. Notably, females also perform much of the farm- and forestry work in rural and farming families which poses a risk for exposure to rodent excreta through handling of e.g., wood, hay, and grain [26, 27]. Also, more women work as small scale entrepreneurs in agricultural tourism, horse-farming etc., while still holding a main occupation outside the farm [26]. Participants in our study were only asked to report their main occupation, thus such agricultural work was hidden.

Although PUUV has been detected in human saliva, implicating a possible risk for person-to-person transmission [9], no increased risks for healthcare workers were found in the present as well as in earlier studies [5].

PUUV infection is correlated to an increased risk for cardiovascular complications i.e., stroke and myocardial infarction [15]. Moreover, some patients suffer from long lasting fatigue. A possible explanation could be impaired lung function [14] but also endocrine deficiencies seem to be common among hospitalized patients with PUUV infection [16]. Some of these deficiencies have been noted for years after recovery from HFRS. It is currently unknown if there are sex-differences in long-term effects of hantavirus-infection. The physical and psychological effects of a potential hypopituitarism after HFRS warrant increased awareness.

A study from Finland, showed higher glomerular filtration rate (GFR) and higher systolic blood pressure in patients who had been diagnosed with PUUV infection 3–7 years earlier, compared to a healthy, seronegative control group [18]. However, in a 10 year follow up, both GFR and blood pressure had normalized and was equal in the groups [17]. Another prospective study published 2009 confirmed the findings relatively early after infection, with higher GFR and systolic blood pressure 6 years after PUUV infection [28]. On the other hand, a Swedish study involving 682 seronegative and 110 seropositive participants in an endemic area in Northern Sweden did not show any differences in blood pressure between the groups [19]. In the present study, there was no significant elevation of blood pressure or GFR, and no higher incidence of diabetes, acute myocardial infarction or cerebrovascular insult were found among the seropositive group i.e., those that had a previous PUUV infection.

For detection of PUUV IgG by ELISA we used the full-length nucleocapsid protein, while a previous study used a truncated nucleocapsid protein [5]. An ELISA based on the full-length protein was more sensitive for detection of PUUV IgM, but the sensitivity for longstanding PUUV IgG has not been assessed [29]. In regard to these circumstances there might be a somewhat higher sensitivity in the present study but that cannot alone explain the increase in PUUV seroprevalence in the population of northern Sweden. Limitations of the present study was the lack of knowledge regarding the year of PUUV infection as well as few reported cases of other illnesses in the material, reflecting the age groups of the study population. Moreover, the studies confirming an association between past PUUV infection and elevated blood pressure and/or renal impairment were mainly conducted on hospitalized patients, indicating a relatively severe form of PUUV infection [18, 28]. It seems likely that the severity of the infection is important for potential sequelae, but long term consequences have been described also after a relatively mild course of PUUV infection [30]. Our study design did not allow for defining the severity of the illness, and additional research of possible comorbidity in hantavirus endemic areas is desirable.

## Conclusions

We have investigated the seroprevalence of Puumala hantavirus that causes haemorrhagic fever with renal syndrome in northern Sweden using a large, randomized and population based cohort.

PUUV antibody presence in serum samples from 1,600 participants was analysed and 13 % were found to be positive. This is substantially higher than previous studies in the same area and might be explained by several PUUV outbreaks in the region, including the large outbreak in 2007. In the older population (65–75 years) as many as one in five individuals have had a PUUV infection and was more common in men than in women.

Smoking, living in rural areas, and owning farmland or forest was identified as risk factors for contracting a PUUV infection. Increased awareness in risk areas, appropriate protection from rodent secreta, and possibly a future vaccine would be of value for the identified high-risk groups.

## Additional file

Additional file 1: Table S1. Self-assessed health and reported disease in relation to Puumala virus (PUUV) seropositivity. (DOCX 73 kb)

## Abbreviations

BMI: Body mass index
CI: Confidence intervals
CKD − EPI: Chronic Kidney Disease Epidemiology Collaboration
ELISA: Enzyme-linked immunosorbent assay
GFR: Glomerular filtration rate
HFRS: Haemorrhagic fever with renal syndrome
HPS: hantavirus pulmonary syndrome
MONICA: Multinational Monitoring of Trends and Determinants in Cardiovascular Disease
OD: Optical density
OR: Odds ratios
PUUV: Puumala hantavirus
WHO: World Health Organisation
