# Supplementary material for: Human Puumala hantavirus infection in northern Sweden; increased seroprevalence and association to risk and health factors
Source: BMC Infect Dis. 2016 Oct 13;16:566. doi: 10.1186/s12879-016-1879-2 (PMC5064900; doi:10.1186/s12879-016-1879-2)
Supplement: Additional file 1: Table S1. — Self-assessed health and reported disease in relation to Puumala virus (PUUV) seropositivity. (DOCX 73 kb) [file 12879_2016_1879_MOESM1_ESM.docx]

**Supplementary table 1. Self-assessed health and reported disease in relation to PUUV seropositivity.**

| Variable (Valid observations) | Subjects within each group n (% of study population) | Seropositity for PUUV n (%) | Multiple logistic regression | |
| --- | --- | --- | --- | --- |
|  |  |  | Odds ratio (OR) | 95% CI^1^ of OR |
| Renal affection^2^ (n = 1563) | 35 (2.2%) | 7 (20.0%) | 0.98 | 0.41-2.30 |
| Hypertension (n = 1596) | 620 (39.0%) | 94 (15.2%) | 0.74 | 0.53−1.01 |
| Asthma^3,4^ (n = 1577) | 161 (10.0%) | 18 (11.2%) | 0.98 | 0.57-1.68 |
| COPD^3,4,5^ (n = 1587) | 21 (1.3%) | 4 (19.0%) | 0.73 | 0.21−2.56 |
| AMI^3,6^ or CVI^3,7^ (n=1600) | 78 (5.0%) | 17 (21.8%) | 1.10 | 0.62−1.95 |
| Diabetes^3^ (n = 1588) | 77 (5.0) | 13 (16.9%) | 1.17 | 0.62−2.19 |
| Unsatisfying health^3^ (n=1572) | 366 (23.0) | 55 (15.0%) | 1.21 | 0.86−1.70 |

^1^CI, Confidence interval, ^2^Renal affection defined as Glomerular filtration rate, GFR <60 mL/min according to the CKD−EPI, ^3^Self-reported, ^4^Also adjusted for smoking, ^5^Chronic obstructive pulmonary disease, ^6^Acute myocardial infarction, ^7^Cerebrovascular insult
